# Recent developments on BMPs and their antagonists in inflammatory bowel diseases

**DOI:** 10.1038/s41420-023-01520-z

**Published:** 2023-07-01

**Authors:** Zhuo Xie, Gaoshi Zhou, Mudan Zhang, Jing Han, Ying Wang, Xiaoling Li, Qirui Wu, Manying Li, Shenghong Zhang

**Affiliations:** Division of Gastroenterology, The First Affiliated Hospital, Sun Yat-sen University, Guangzhou, P. R. China

**Keywords:** Inflammatory bowel disease, Molecular biology

## Abstract

Inflammatory bowel diseases (IBDs), including ulcerative colitis, and Crohn’s disease, are intestinal disorders characterized by chronic relapsing inflammation. A large proportion of patients with IBD will progress to develop colitis-associated colorectal cancer due to the chronic intestinal inflammation. Biologic agents that target tumour necrosis factor-α, integrin α4β7, and interleukin (IL)12/23p40 have been more successful than conventional therapies in treating IBD. However, drug intolerance and loss of response are serious drawbacks of current biologics, necessitating the development of novel drugs that target specific pathways in IBD pathogenesis. One promising group of candidate molecules are bone morphogenetic proteins (BMPs), members of the TGF-β family involved in regulating morphogenesis, homeostasis, stemness, and inflammatory responses in the gastrointestinal tract. Also worth examining are BMP antagonists, major regulators of these proteins. Evidence has shown that BMPs (especially BMP4/6/7) and BMP antagonists (especially Gremlin1 and follistatin-like protein 1) play essential roles in IBD pathogenesis. In this review, we provide an updated overview on the involvement of BMPs and BMP antagonists in IBD pathogenesis and in regulating the fate of intestinal stem cells. We also described the expression patterns of BMPs and BMP antagonists along the intestinal crypt-villus axis. Lastly, we synthesized available research on negative regulators of BMP signalling. This review summarizes recent developments on BMPs and BMP antagonists in IBD pathogenesis, which provides novel insights into future therapeutic strategies.

## Facts

- Inflammatory bowel diseases (IBDs) are intestinal disorders characterized by chronic relapsing inflammation; novel biologics or drugs are needed to optimize treatment outcome.
- BMPs can modulate morphogenesis, homeostasis, stem cells and inflammatory responses in the gastrointestinal tract.
- BMPs and their antagonists have distinct expression patterns in the villus-crypt axis.
- Recent developments on BMPs and BMP antagonists in IBD pathogenesis offer novel insights into future therapeutic strategies.

## Open questions

- What’s the precise function of other BMPs and BMP antagonists that have not been well studied?
- What’s the specific role of BMPs and BMP antagonists in the intestinal stem cell niche of IBD patients?
- In addition to the five well-known extracellular BMP antagonists, will other negative inhibitors of BMPs play important roles in IBD as well?

## Introduction

The aetiology of inflammatory bowel diseases (IBDs) involves susceptibility genes, environmental impacts on the microbiome and abnormal immune responses [1]. Two common IBDs include ulcerative colitis (UC) and Crohn’s disease (CD). Chronic inflammation during IBD increases the risk of developing colitis-associated colorectal cancer (CAC) [2].

Biologic agents to treat IBDs are more effective than conventional therapy. Common options include anti-tumour necrosis factor-α (infliximab and adalimumab), anti-integrin α4β7 (vedolizumab), and anti-IL12/23p40 (ustekinumab) agents. Nevertheless, these compounds have major drawbacks, including drug intolerance among patients [3]. Novel biologics are thus needed to optimize treatment outcome.

Abnormal bone morphogenetic protein (BMP) signalling and disruption of intestinal homeostasis are increasingly recognized as components of IBD pathogenesis. Originally, BMPs were understood only as regulators of bone and cartilage formation, but now they are also known to modulate morphogenesis, homeostasis, stem cells, and inflammatory responses in other tissues, including the gastrointestinal tract [4, 5].

Notable negative regulators of BMP action are BMP antagonists, which prevent the BMPs from binding to their cognate receptors at the cell surface [6]. BMP antagonists are characterized by cysteine-rich domains and are divided into five types based on the distance of cysteine residues. The first is the DAN family, comprising Gremlin (Grem1), sclerostin (SOST), uterine sensitization-associated gene 1 (USAG-1), Dante (Dte), protein related to DAN or Cerberus (PRDC/Grem2) and Coco. The other four types are Noggin (Nog), Chordin (Chrd), follistatin (FST) and twisted gastrulation (Twsg) [6, 7].

This review summarized current research on BMP signalling in the intestines, focusing on the roles of BMP2, BMP3, BMP4, BMP6, BMP7 and BMP9 in intestinal inflammation and IBD pathogenesis. We also discussed the involvement of BMP antagonists, including Grem1, SOST, Nog and FST, in IBD. Our third topic of focus was the involvement of BMPs and BMP antagonists in intestinal stem cell (ISC) fate. Finally, we covered expression patterns of BMPs and BMP antagonists along the intestinal crypt-villus axis, while also highlighting other negative regulators of BMP signalling.

## BMP signalling in the intestines

**Fig. 1:**
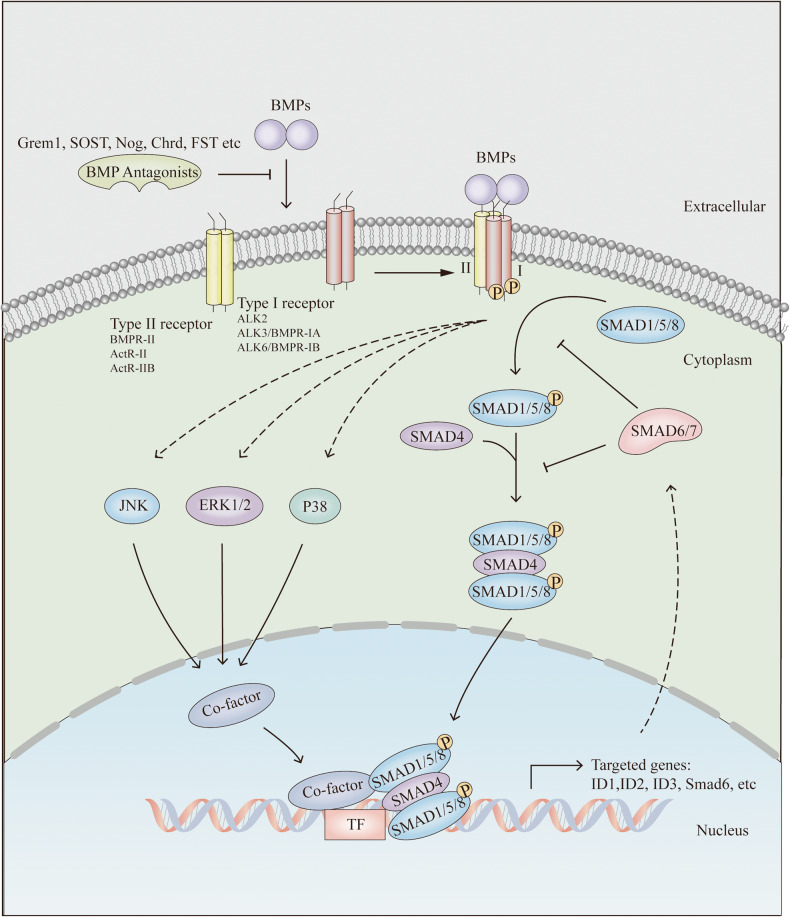
BMP signalling in the intestines. Activities of BMP signalling in the intestines can be inhibited by a group of antagonists, including the DAN family members (Grem1, SOST, Nog, Chrd, FST and Twsg. As dimers, BMPs can bind to two distinct receptor types (I and II), both with serine/threonine kinase activity at the plasma membrane. The canonical Smad pathway is triggered when extracellular BMPs bind to a type II receptor, a receptor complex of type II and type I receptor is formed, leading to the phosphorylation and activation of type I by type II receptors. The activated type I receptor then recruits and phosphorylates downstream Smad1/5/8, two of which forms a complex with one Smad4. This Smad complex is transported to the nucleus and regulates the transcription of downstream ID1/2/3 and Smad6. Smad6/7 are inhibitors of Smad1/5/8 phosphorylation and complex formation between p-Smad1/5/8 and Smad4. The non-Smad signalling pathway is also triggered upon BMPs binding to receptors. The most notable pathways involve MAPK (p38, JNK, ERK1/2), which activate downstream co-factors and then regulates target gene expression with Smad.

BMP signalling is critical to development, stem cell homeostasis and intestinal diseases [5, 8]. Enabled through differential expression and localization of ligands, receptors, and antagonists, BMP signals exhibit a polarized gradient along the crypt-villus axis, being highest at the top of the villus [9]. Activities of BMP signalling in the intestines can be inhibited by a group of antagonists, including the DAN family members (Grem1 [10], SOST [11]), Nog [12], Chrd [9], FST [13] and Twsg [14] (Fig. 1).

As dimers, BMPs can bind to two distinct receptor types (I and II), both with serine/threonine kinase activity at the plasma membrane [15] (Fig. 1). Type I receptors include activin receptor-like kinase (ALK)-2, ALK-3 (BMPR-IA), and ALK-6 (BMPR-IB) (Fig. 1). Type II receptors are further subdivided into three categories: BMP type II receptor (BMPR-II), activin type II receptor (ActR-II), and activin type IIB receptors (ActR-IIB) (Fig. 1). Of the 15 BMP types identified in humans [16], BMP2 [17] and BMP4 [18] are dominant in the intestines.

BMPs evoke downstream responses via canonical Smad [19] and non-Smad pathways [20] (Fig. 1). Smad proteins can be divided into receptor-regulated Smad (R-Smad, Smad1/5/8), common Smad (Co-Smad, Smad4), and inhibitory Smad (I-Smad, Smad6/7) [21] (Fig. 1).

The canonical Smad pathway is triggered when extracellular BMPs bind to a type II receptor, a receptor complex of type II and type I receptor is formed, leading to the phosphorylation and activation of type I by type II receptors (Fig. 1). The activated type I receptor then recruits and phosphorylates downstream Smad1/5/8 (R-Smad), two of which forms a complex with one Smad4 (Co-Smad; Fig. 1). This Smad complex is transported to the nucleus and regulates the transcription of downstream inhibitor of DNA-binding (ID) proteins (ID1 [22], ID2 [23] and ID3 [24]) and inhibitory Smads (Smad6) [19] (Fig. 1). Significantly, ID proteins are identified as functional markers for ISCs [22–24]. Smad6/7 (I-Smad) are inhibitors of Smad1/5/8 phosphorylation and complex formation between p-Smad1/5/8 and Smad4 [21] (Fig. 1).

The non-Smad signalling pathway is also triggered upon BMPs binding to receptors. The most notable pathways involve mitogen-activated protein kinases (MAPK) (p38, c-Jun amino-terminal kinase (JNK), and extracellular signal-regulated kinases (ERK1/2)), which activate downstream co-factors and then regulates target gene expression with Smad [20] (Fig. 1).

## Role of BMPs in IBD pathogenesis

Several studies have shown that BMPs play critical roles in colitis and IBD pathogenesis. Here, we review the latest research on BMPs that may serve as potential targets for IBD treatment. BMPs function via binding to BMP receptors with different forces. BMP2 and BMP4 bind strongly to ALK3 and ALK6, whereas BMP6 and BMP7 have high affinities for ALK2 but low affinities for ALK6 [5] (Table 1). BMP9 binds to ALK1 and ALK2 [25] (Table 1).

**Table 1:** Role of BMPs in IBD pathogenesis.

BMPs	Binding	Function	Refs.
BMP2	High affinity with ALK3/BMPRIA and ALK6/BMPRIB	Inhibit proliferation, induce apoptosis, and mediate angiogenesis in vitro	[17, 35, 39]
BMP3	/	Antagonise BMP2; methylated BMP3 served as a faecal biomarker to distinguish IBD patients with or without colorectal dysplasia and CAC	[42–44]
BMP4	High affinity with ALK3/BMPRIA and ALK6/BMPRIB	Exert an anti-inflammatory role and play a critical role in the ISCs in the pathogenesis of IBD	[24, 26–28]
BMP6	High affinity with ALK2, weakly with ALK6/BMPRIB, hepcidin	Anti-BMP6 reagents attenuate intestinal inflammation in the DSS-induced colitis mice and correct the anaemia of IBD	[29–32, 34, 35]
BMP7	High affinity with ALK2, weakly with ALK6/BMPRIB	Alleviate inflammation in TNBS-induced colitis and prevent intestinal fibrosis in the process of IBD	[38–41]
BMP9/GDF2	ALK1 and ALK2	BMP9 crosstalk with several pathways is associated with intestinal inflammatory responses and may impact the pathogenesis of IBD	[47–49]

The binding targets and function of BMP2, BMP3, BMP4, BMP6, BMP7, and BMP9.

### BMP4

Despite targeting the epithelium, BMP4 is localized in mesenchymal cells expressing α-smooth muscle actin [26]. A major function of BMP4 is mitigating colonic inflammation and maintaining intestinal homeostasis [26]. Deletion of epithelial *Bmpr1a* enhances BMP4 in dextran sodium sulphate (DSS)-induced colitis, whereas inflammatory cytokines TNF-α and IL-1β both inhibit BMP4 [26]. Recent research on DSS-induced colitis found that expression patterns were associated with disease progression; specifically, BMP4 and Smad4 expression in the crypt was upregulated during early-stage DSS-induced colitis and downregulated during the late stage [24]. The disease can be ameliorated through treatment with exogenous BMP4 recombinant protein, which targets an ID3 inhibitor to increase epithelial proliferation and maintain Lgr5+ intestinal stem cells [24]. However, transgenic overexpression of BMP4 ligands in the intestinal crypt-villus axis inhibits proliferation, accelerates terminal differentiation, and impairs intestinal regeneration in DSS-induced colitis [27]. Moreover, BMP4 inhibition in intestinal stromal cells promotes ISC proliferation and maintenance of intestinal homeostasis [28]. Thus, multiple factors (e.g., localization, concentration, targets) clearly influence whether BMP4 exerts anti-proliferative or pro-proliferative effects on the intestinal epithelium. Nevertheless, we can conclude that BMP4 exerts an anti-inflammatory effect and is critical to ISCs in IBD pathogenesis (Table 1).

### BMP6

BMP6 function as a regulator of hepcidin expression and iron metabolism [29]. Circulating iron levels modulate BMPs expression, and lack of BMP6 causes iron overload [30, 31]. Hepcidin and iron homeostasis participate in the pathogenesis of intestinal inflammation [32]. Anti-BMP6 reagents attenuate intestinal inflammation in DSS-induced colitis mice and mitigate IBD anaemia [33] (Table 1). The mechanism of actions appears to be interacting with IL-6 expression and downregulating hepcidin expression [34]. BMP6 thus plays an essential role in the inflammatory response and iron homeostasis of IBD.

Interestingly, a recent study identified the mechanism of BMP6-regulated angiogenesis as modulating vascular endothelial growth factor receptor 2 via Hippo/TAZ signalling [35]. This finding opens a new arena of research regarding the influence of BMP6 on angiogenesis, an important pathological characteristic of IBD that contributes to the disease’s initiation and perpetuation [36, 37].

### BMP7

Previous studies have revealed that although BMP7 has anti-inflammatory effects, its levels significantly decrease in the acute phase of TNBS-induced colitis [38, 39]. However, experiments with rat models of colitis demonstrated the prominence of exogenous BMP7 in lowering pro-inflammatory cytokine production (especially IL-6) and thus protecting mucosa [38] (Table 1). In addition, while BMP7 also decreased significantly in the acute phase among rats, exogenous BMP7 treatment elevated BMP signalling through influencing the expression of several BMPs (BMP2 and BMP4), connective tissue growth factor (CTGF), Nog, and BR-Smad (Smad3 and Smad4) [39].

Importantly, BMP7 expression increased in stenotic intestinal tissue of Crohn’s disease patients [40]. As an antagonist of TGF-β1, BMP7 can prevent epithelial-to-mesenchymal transition (EMT) and induced intestinal fibrosis [41] (Table 1). These findings collectively show that BMP7 is a promising therapeutic candidate for IBD, given its ability to alleviate intestinal inflammation and prevent intestinal fibrosis.

### Other BMPs that may be involved in IBD pathogenesis

BMP2 is expressed in the mature colonocytes of the epithelial surface of the normal colon and it influences the same cells that produce in an autocrine manner [17], but its expression increases significantly during the acute phase of 2,4,6-trinitrobenzene sulfonic acid (TNBS)-induced colitis [39]. In vitro, BMP2 inhibits proliferation and induces apoptosis, decreasing the expression of the cyclin proliferating cell nuclear antigen (PCNA) while increasing caspase 3 and β-catenin expression [17] (Table 1). Additionally, BMP2 is involved in VEGF-mediated endothelial sprouting through regulating delta-like canonical Notch ligand 4 (DLL4) [35]. Taken together, these findings suggest that BMP2 plays a role in the pathogenesis of TNBS colitis, but the exact mechanism remains unclear.

BMP3 competes with BMP2 for essential components in TGF-beta/activin and BMP pathways [42] (Table 1). In colorectal cancer, BMP3 is frequently inactivated via hypermethylation, and its active form functions as a tumour suppressor [43]. Methylated BMP3 is widely acknowledged as a faecal biomarker that can specifically discriminate between IBD patients with or without colorectal dysplasia and CAC. In a prospective blinded study carried out by Kisiel [44], buffered stool-extracted DNA from 19 IBD cases with dysplasia or CAC and 35 IBD controls without dysplasia or CAC were analysed; BMP3 showed a high association in stools for dysplasia and CAC.

Additionally, in a study done by Johnson at a single centre in 2 blinded phases, BMP3 methylation was higher in mucosae and stool from 29 IBD patients with dysplasia compared to that of 44 matched IBD controls [45].

Recently, in an analysis of faecal samples from 3 independent studies of 332 patients with IBD, levels of methylated BMP3 demonstrated a high sensitivity and specificity for identification of colorectal dysplasia and CAC in IBD patients [46].

In conclusion, these studies suggest that BMP3 has the potential to be a non-invasive faecal biomarker for early detection for colorectal dysplasia and CAC in IBD patients.

BMP9, also called growth differentiation factor 2, regulates phosphoinositide-3-kinase (PI3K)/AKT signalling [47, 48] independent of Smad proteins. Through upregulating PTEN, BMP9 inhibits PI3K/AKT signalling, inhibiting proliferation and inducing apoptosis in colon cancer cells [47]. A recent study further revealed that combining BMP9 with ALK1 inactivates the PI3K/AKT pathway, suppressing osteosarcoma proliferation and metastasis [48].

In addition to inhibiting PI3K/AKT signalling, BMP9 activates multiple MAPKs through phosphorylation [49], including ERK1/2, p38, and JNK1/2. Therefore, BMP9 crosstalk is associated with intestinal inflammatory responses and may affect IBD pathogenesis (Table 1).

## Role of BMP antagonists in IBD pathogenesis

The activities of BMPs are inhibited by a family of extracellular secreted BMP antagonists. Here, we review the latest discoveries on the role of BMP antagonists in IBD pathogenesis.

### Gremlin (Grem1)

Grem1 binds to BMP2, BMP4, and BMP7 with high affinity, preventing their interaction with BMPRs [50] (Table 2). Because it is upregulated in human IBD and mouse DSS-induced colitis tissues [27], targeting the Grem1-BMP pathway has therapeutic potential for patients with IBD. Cumulative stromal Grem1 secretion rapidly and continuously inhibits BMP signalling in colonic ulceration or impairment, and ectopic epithelial Grem1 expression accelerates intestinal epithelial regeneration [27] (Table 2). Transcriptome profiling of colonic biopsies revealed that UC patients with a long disease duration (and thus, higher risk of developing CAC) had significantly lower Gem1 expression than patients with short disease duration [51]. In UC colonic mucosa, mesenchymal Grem1 expression is strongly associated with increased proliferation and aberrant differentiation of tissue-resident mesenchymal stem cells [52] (Table 2). In addition, aberrant epithelial Grem1 expression contributes to colonic tumorigenesis [53]. Overall, the available data supports Grem1 as a critical BMP antagonist in epithelial regeneration and differentiation of IBD.

**Table 2:** Role of BMP antagonists in IBD pathogenesis.

BMP antagonists	Binding targets	Main functions	Refs.
Gremlin (Grem1)	High affinity with BMP2, BMP4, and BMP 7	Ectopic epithelial expression of Grem1 can drastically accelerate intestinal epithelial regeneration; mesenchymal Grem1 promotes intestinal proliferation and regulates aberrant differentiation of tissue-resident mesenchymal stem cells in UC colonic mucosa.	[27, 50–53]
Sclerostin (SOST)	High affinity with BMP6 and BMP7 and low affinity with BMP2 and BMP4	Act as the novel biomarkers to predict the presence of axial joint inflammation in IBD patients	[54–56]
Noggin (Nog)	High affinity with BMP2 and BMP4 and low affinity with BMP7	Nog is involved in the colonic organoid differentiation in humans and mice; overexpression of Nog in the developing neurons of increases the ultimate number of enteric neurons and aggravates intestinal inflammation in the dextran sodium sulphate (DSS)/2,4,6-trinitrobenzene sulfonic acid (TNBS)-induced colitis	[17, 18, 39, 59–61]
Follistatin (FST)	High affinity with BMP4 and BMP6 and low affinity with BMP7	Administration with FST promotes tissue repair and alleviates the severity of DSS colitis, TNBS colitis, and IL-10 gene deficiency-induced spontaneous colitis; FSTL1 regulates macrophage polarisation and exacerbates DSS-induced colitis	[63–66]

### Sclerostin (SOST)

SOST is a high-affinity ligand of BMP6/7 and BMP antagonist, although it only weakly binds to BMP2/4 [54] (Table 2). The early diagnosis of IBD-associated spondyloarthritis (SpA/IBD) in IBD patients is essential; however, there is a lack of serum biomarkers to demonstrate joint inflammation. In a study done by Luchetti [55], serum levels of SOST were substantially lower in IBD patients with axial SpA and were associated with the articular symptoms compared to that of IBD patients without axial SpA and healthy controls. Serum levels of SOST and anti-SOST antibodies may serve as novel potential biomarkers to predict the presence of axial joint inflammation in IBD patients [55, 56] (Table 2).

### Noggin

The glycoprotein Nog is a BMP antagonist with high affinity for BMP2/4 and low affinity for BMP7 [57, 58], expressed in mature colonic epithelial cells [17] (Table 2). Treatment of mice with recombinant Nog inhibits proliferation and apoptosis [17]. In both humans and mice, Nog is implicated in colonic organoid differentiation [59] (Table 2). Indeed, *Nog*-transgenic mice exhibit numerous ectopic crypt villus units, suggesting that inactivation of BMP signalling facilitates de novo crypt formation [18].

Nog expression is elevated in the acute stage of experimental IBD models and decreases significantly with BMP7 therapy [39]. Nog’s effects may be related to the enteric nervous system, which has also been implicated in IBD given its role in coordinating digestive processes and gastrointestinal homeostasis [60]. Nog overexpression in developing neurons of transgenic mice increased the number of enteric neurons and significantly worsened intestinal inflammation in DSS-/TNBS-induced colitis [61] (Table 2).

### Follistatin

FST is a single-chain glycosylated protein that binds and neutralizes BMPs [62]. Studies have found that FST has high affinity for BMP 4/6, but low affinity for BMP7 [63] (Table 2). In mouse TNBS-induced colitis models, FST administration markedly increased the mice survival rate and decrease plasma IL-6 levels [64]. FST treatment also promotes tissue repair and alleviates the severity of DSS colitis, TNBS colitis, and IL-10 gene deficiency-induced spontaneous colitis [64] (Table 2).

The pro-inflammatory cytokine follistatin-like protein 1 (FSTL1) is a member of the FST class that is upregulated in active colitis of human and mice [65]. Serum FSTL1 levels were found to be extremely higher in UC patients than in normal controls [66]. Importantly, FSTL1 greatly enhances the production of other inflammatory cytokines through facilitating M1 pro-inflammatory polarization and inhibiting M2 anti-inflammatory polarization of macrophages; these activities then serve to aggravate DSS-induced colitis [65] (Table 2).

## ISC fate is related to expression patterns of BMPs and BMP antagonists along the intestinal crypt-villus axis

**Fig. 2:**
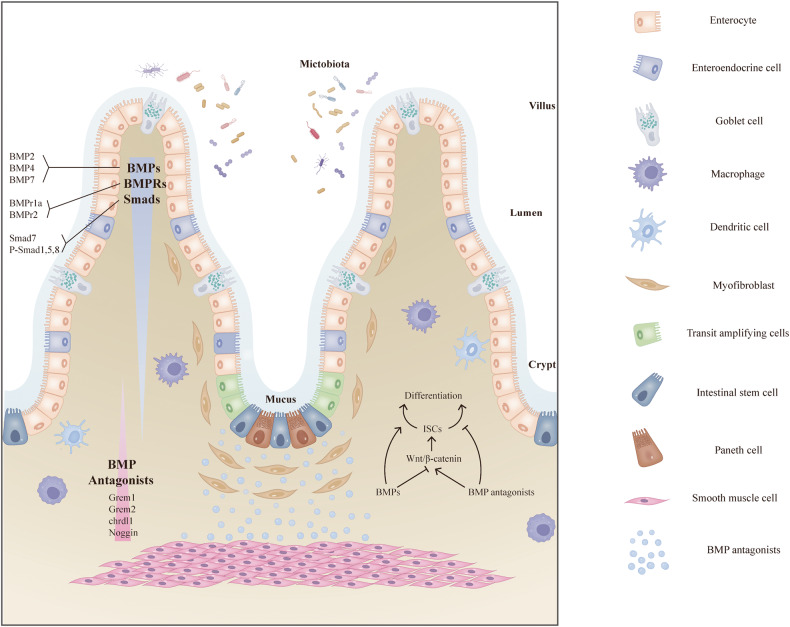
ISC fate is related to expression patterns of BMPs and BMP antagonists along the intestinal crypt-villus axis. In the ISC compartment, BMP signalling inhibits ISC activation and supports intestinal transit, amplifying cell differentiation into multiple mature cell lineages. Through inhibiting Wnt/β-catenin pathways, BMP signalling suppresses ISC self-renewal. Importantly, epithelial BMP signalling directly restricts Lgr5^+^ stem cells via Smad-mediated transcriptional inhibition of multiple signature ISC genes, independent of Wnt signalling. BMPs and their antagonists have distinct expression patterns in the villus-crypt axis. BMPs, including BMP2/4/7 originate from stromal cells below the epithelium and are highly expressed in the upper crypts before gradually dispersing to the bottom crypts. The intestinal expression patterns of BMP receptors (e.g., BMPr1a, BMPR2) and Smad proteins (e.g., Smad7, phosphorylated (p)-Smad1/5/8) also have a gradient distribution pattern along the crypt-villus axis. In contrast, BMP antagonists Grem1, Grem2, Chrdl1 and Nog are highly expressed at the bottom of the crypt and barely expressed in the villus. These proteins are secreted from mesenchyme-derived tissue (myofibroblasts and smooth muscle cells) and make contributions to the ISC niche via activating Wnt-beta-catenin signalling and suppressing intestinal differentiation.

The intestinal epithelium contains multiple cell types and is renewed every 4–5 days [67]. The self-renewal and differentiation of ISCs maintains intestinal homeostasis, while the ISCs themselves are regulated by a unique niche environment [68]. Multiple pathways modulate stemness within the ISC niche, including the BMP, Wnt and Hh pathways [69]. In the ISC compartment, BMP signalling inhibits ISC activation and supports intestinal transit, amplifying cell differentiation into multiple mature cell lineages [70, 71] (Fig. 2). Through inhibiting Wnt/β-catenin pathways, BMP signalling suppresses ISC self-renewal [72] (Fig. 2). Importantly, epithelial BMP signalling directly restricts Lgr5^+^ stem cells via Smad-mediated transcriptional inhibition of multiple signature ISC genes (e.g., *Lgr5*, *Sox9* and *Tnfrsf19*); this inhibition occurs independent of Wnt signalling [73] (Fig. 2).

BMPs and their antagonists have distinct expression patterns in the villus-crypt axis. BMPs, including BMP2/4/7 originate from stromal cells below the epithelium and are highly expressed in the upper crypts before gradually dispersing to the bottom crypts [9, 71] (Fig. 2). The intestinal expression patterns of BMP receptors (e.g., BMPr1a [72], BMPR2 [9]) and Smad proteins (e.g., Smad7 [9], phosphorylated (p)-Smad 1/5/8 [72]) also have a gradient distribution pattern along the crypt-villus axis (Fig. 2). BMPr1a and P-Smad1/5/8 are express in ISCs [72]. In contrast, BMP antagonists Grem1, Grem2, Chrdl1, and Nog are highly expressed at the bottom of the crypt and barely expressed in the villus (Fig. 2). These proteins are secreted from mesenchyme-derived tissue (myofibroblasts and smooth muscle cells) and make contributions to the ISC niche via activating Wnt-beta-catenin signalling and suppressing intestinal differentiation [9, 71]. Further studies are clearly necessary to expand on the role of BMP antagonists in the ISC niche of IBD patients, as the results should offer novel insights into future therapeutic strategies.

## Other negative regulators of BMP signalling

In addition to the five well-known extracellular BMP antagonists (Table 3), other mechanisms can suppress BMP signalling. In the plasma membrane, non-signalling BMP pseudoreceptors, like BMP and activin membrane-bound inhibitor (BAMBI), block BMPs through interactions with BMPRIA and BMPRIB [74] (Table 3). In the cytoplasm, BMP activity can be blocked by numerous mechanisms. First, BMPRI/ALK2 phosphorylation can be suppressed by FK-binding protein-12 (FKBP12) [75], while I-Smads (Smad6/7) can repress Smad formation and activation [21] (Table 3). MicroRNA silencing of BMP components (such as disruption of BMPR1A by miR-885-3p [76]), direct binding with BMP-specific Smad1/5 and Smad4, and interruption of BMP signalling by Smad-binding proteins Ski [77] and transducer of Erb B-2 (Tob) [78] are other documented mechanisms (Table 3). Finally, BMP activity is inhibited through Smad1/5 ubiquitination and degradation by Smad-specific E3-ubiquitin ligases, including Smad ubiquitination regulatory factor 1 (Smurf1) [79] and Smurf 2 [80] (Table 3). In the nucleus, methylation or hypermethylation are the mechanisms of BMP downregulation [81] (Table 3).

**Table 3:** Negative regulators of BMP signalling.

Localisation	Types	Classification	Name	Function	Refs.
Extracellular matrix	BMP antagonists	1) The Dan family	Grem1	Prevent BMP signalling by binding BMPs extracellularly	[9–14]
			SOST		
			USAG-1		
			Dte		
			PRDC/Grem2		
		2) Noggin	Nog		
		3) Chordin	Chrd		
		4) Follistatin	FST		
		5) Twisted gastrulation	Twsg		
Plasma membrane	Nonsignalling BMP pseudoreceptors		BAMBI	Block BMPs through interactions with BMPRIA and BMPRIB	[74]
Cytoplasm	BMPR inhibitor		FKBP12	Suppress BMPRI/ALK2 phosphorylation	[75]
	Inhibitory-Smads		Smad6/7	Repression of the formation and activation of Smads	[21]
	MicroRNAs		MiR-885-3p	Silencing of BMP components	[76]
	Smad-binding protein		Ski and Tob	Directly binds with BMP-specific Smad1/5 and Smad4 and interrupts BMP signalling	[77, 78]
	Ubiquitination and degradation of Smads		Smurf1 and 2	Ubiquitinate and degrade Smad1/5	[79, 80]
Nucleus	Methylation or hypermethylation		Methylated BMPs	Downregulate BMPs via gene methylation or hypermethylation	[81]

Clearly, BMP signalling is under a complex regulatory system in both extracellular and intracellular spaces. However, further research is needed to uncover exactly how these negative regulators of BMPs function in IBD.

## Discussion

Originally knowns as regulator of bone and cartilage formation, BMPs can also modulate morphogenesis, homeostasis, stem cells, and inflammatory responses in the gastrointestinal tract. Notable BMP antagonists are divided into five types. The first is the DAN family, comprising Gremlin (Grem1), sclerostin (SOST), uterine sensitization-associated gene 1 (USAG-1), Dante (Dte), protein related to DAN or Cerberus (PRDC/Grem2) and Coco. The other four types are Noggin (Nog), Chordin (Chrd), follistatin (FST) and twisted gastrulation (Twsg). Abnormal BMP signalling and disruption of intestinal homeostasis are increasingly recognized as components of IBD pathogenesis.

This review highlights new insight into the role of BMPs and BMP antagonists in IBD pathogenesis. In addition, available research strongly suggests that the BMP signalling pathway has potential as a therapeutic target or novel biomarker for IBD/CAC. Importantly, BMP4, BMP6 and BMP7 have been studied to play essential roles in IBD. BMP4 exerts an anti- inflammatory role and plays a critical role in the ISCs in the pathogenesis of IBD; Anti-BMP6 reagents attenuate intestinal inflammation in the DSS-induced colitis mice and correct the anaemia of IBD; BMP7 alleviates inflammation in TNBS-induced colitis and prevent intestinal fibrosis in the process of IBD. Methylated BMP3 has been suggested as a potential biomarker in stool DNA surveillance testing for CAC surveillance in IBD patients. Regarding BMP antagonists, Grem1, SOST, Nog and FST/FSTL1 have been reported to involve in IBD pathogenesis or serve as biomarkers in IBD patients. For instance, ectopic epithelial expression of Grem1 can dramatically accelerates the intestinal epithelial regeneration; Mesenchymal Grem1 promotes intestinal proliferation and regulates aberrant differentiation of tissue-resident mesenchymal stem cells in UC colonic mucosa; SOST acts as the novel biomarkers to predict the presence of axial joint inflammation in IBD patients; Nog is involved in the colonic organoid differentiation of human and mice; Overexpression of Nog in the developing neurons of increases the ultimate number of enteric neurons and aggravates intestinal inflammation in the DSS-/TNBS-induced colitis; Administration with FST promotes tissue repair and alleviates the severity of DSS colitis, TNBS colitis and IL-10 gene deficiency-induced spontaneous colitis; FSTL1 regulates macrophage polarization and exacerbates DSS -induced colitis.

However, many unknowns remain. For example, we do not know how IBD pathological processes will be affected by combined intervention of two or more BMPs and BMP antagonists with new or optimized function. Beyond the known BMP antagonists, other negative regulators of BMP signalling and their involvement in intestinal inflammation or colitis remain understudied. Even within BMPs and BMP antagonists, numerous members of both groups are poorly understood, particularly with respect to intestinal inflammation or IBD. BMP3 and SOST are potential biomarkers for IBD with dysplasia or CAC and axial SpA/IBD, respectively; however, other BMP-relevant molecules may also have the potential to be novel biomarkers in IBD patients.

Current IBD treatment biologics mainly include anti-tumour necrosis factor-α (infliximab and adalimumab), anti-integrin α4β7 (vedolizumab), and anti-IL12/23p40 (ustekinumab) agents. Additionally, there are emerging IBD treatment biological agents that have achieved great clinical efficacy, including JAK1/3 inhibitors and anti-IL6 agents [82]. In this review, we concluded that anti-BMP6 reagents, exogenous BMP7 and FST all have impacts on decreasing IBD-associated pro-inflammatory cytokines, especially IL-6 which attenuates DSS-induced mice colitis. Stem cell transplantation has shown favourable effects in IBD clinical trials [83]. The intestinal organoid therapy for UC is now under estimated in human clinical trial in Japan and is progressing well [84]. Therefore, regulating the ISCs is also a potential therapeutic approach to IBD treatment. BMP4 and Grem1 were revealed to regulate the ISC function, which suggests BMP signalling is critical to the regulation of ISC fate [72, 73]. A worthwhile direction of research is exploring the impact of modulating BMP signalling on intestinal organoid cultures and mesenchymal stem cell treatments for IBD. These data highlight the significance of conducting BMP-related research and the potential contribution to the clinical treatment of IBD. As demand for novel IBD treatments increases, targeting BMP signalling to manipulate the regulation of multiple cell types will help to accelerate the development of additional therapeutic options.
